# Utility of Electroencephalograms for Enhancing Clinical Care and Rehabilitation of Children with Acquired Brain Injury

**DOI:** 10.3390/ijerph21111466

**Published:** 2024-11-02

**Authors:** Keren Politi, Patrice L. Weiss, Kfir Givony, Elana Zion Golumbic

**Affiliations:** 1 ALYN Hospital, Jerusalem 91090, Israel; 2 Helmsley Pediatric & Adolescent Rehabilitation Research Center, ALYN Hospital, Jerusalem 91090, Israel; tamarw@alyn.org (P.L.W.); kfirgivony@gmail.com (K.G.); 3 Department of Occupational Therapy, University of Haifa, Haifa 3498838, Israel; 4 The Faculty of Medicine, Hebrew University of Jerusalem, Jerusalem 91120, Israel; 5 The Gonda Center for Multidisciplinary Brain Research, Bar Ilan University, Ramat Gan 5290002, Israel; elana.zion-golumbic@biu.ac.il

**Keywords:** electroencephalography, children, acquired brain injury, rehabilitation, cognitive and motor impairment

## Abstract

The objective of this literature review was to present evidence from recent studies and applications focused on employing electroencephalogram (EEG) monitoring and methodological approaches during the rehabilitation of children with acquired brain injuries and their related effects. We describe acquired brain injury (ABI) as one of the most common reasons for cognitive and motor disabilities in children that significantly impact their safety, independence, and overall quality of life. These disabilities manifest as dysfunctions in cognition, gait, balance, upper-limb coordination, and hand dexterity. Rehabilitation treatment aims to restore and optimize these impaired functions to help children regain autonomy and enhance their quality of life. Recent advancements in monitoring technologies such as EEG measurements are increasingly playing a role in clinical diagnosis and management. A significant advantage of incorporating EEG technology in pediatric rehabilitation is its ability to provide continuous and objective quantitative monitoring of a child’s neurological status. This allows for the real-time assessment of improvement or deterioration in brain function, including, but not limited to, a significant impact on motor function. EEG monitoring enables healthcare providers to tailor and adjust interventions—both pharmacological and rehabilitative—based on the child’s current neurological status.

## 1. Introduction

Acquired brain injury (ABI) is the leading cause of death and neurological impairment in children after infancy. The primary causes of ABI include trauma (e.g., motor vehicle accidents, injuries, and falls), infections (e.g., meningitis, encephalitis), and diseases (e.g., stroke and tumors). Traumatic brain injury (TBI), the most prevalent form of ABI, poses a significant public health concern. The incidence of TBI is notably high, with nearly 300 out of every 100,000 children under the age of 18 years in North America experiencing a TBI annually [1,2,3].

Children who sustain moderate to severe TBI are at an increased risk of chronic neurological complications, such as epilepsy, developmental delays, cognitive and meta-cognitive impairments, and behavioral problems [1,2,3]. Evidence indicates that pediatric TBI patients are susceptible to secondary attention deficit and hyperactivity disorder (S-ADHD) and other psychiatric disorders [4,5]. The functional impact of these complications varies depending on the child’s age at the time of injury and the severity of the injury [4,6,7,8,9].

**Figure 1:**
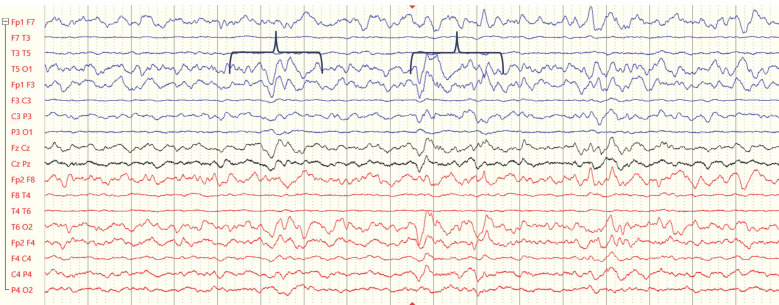
Example EEG recordings from a 4-year-old child during the subacute period following ABI (traces from electrodes over the right and left hemispheres are presented in red and blue, respectively). Black markers indicate epochs with increased theta and delta activity.

Neuronal injury resulting from ABI can be assessed and characterized using electroencephalography, a non-invasive method for recording neural activity that provides millisecond-precision information about brain dynamics. Several EEG measures can be indicative of the patient’s current neurological state and prognosis. One prominent example is the spectral makeup of the EEG, commonly referred to as quantitative EEG (qEEG), which involves the decomposition of the EEG signal into canonical frequency bands that are associated with different functional characteristics. These typically include delta (1–3 Hz), theta (4–7 Hz), alpha (8–12 Hz), beta (13–30 Hz), and gamma (30–100 Hz) bands. Many of these spectral features show prominent abnormalities in TBI patients. For example, increased theta and delta activity are commonly observed in patients with TBI (Figure 1), particularly in those with severe injuries [10]. The prominence of these slow waves can be indicative of cortical dysfunction and are often correlated with poor outcomes, such as levels of unconsciousness and reduced motor function [10,11]. Other qEEG characteristics of TBI include reduced alpha power and increased beta power, frequencies that are often associated with a state of relaxation and active thinking, respectively. This pattern has been suggested to reflect disrupted cortical connectivity and compensatory mechanisms in response to injury [12,13]. The relationship between frequency bands and their cross-frequency coupling can also yield important insights as to the patient’s clinical state [14]. Some studies have found that measures of functional connectivity and spectral features can be helpful not only in the diagnosis of acute brain injury, but also in the assessment of its severity and long-term outcomes [15,16,17,18]. Besides the direct neural damage caused by TBI, the injury often leads to epileptogenesis, and many patients develop post-traumatic epilepsy or exhibit epileptic electrographic features that are visible only through EEG [19].

Despite the direct ‘window into the brain’ that EEGs afford, and the fact that using them is a relatively inexpensive method, to date, EEGs are not yet sufficiently incorporated as a clinical tool for patient evaluation during the chronic stages following ABI, as well as throughout the rehabilitation process [10]. We contend that periodic EEG monitoring following ABI could make a critical contribution to the diagnosis, treatment, and follow-up of neurological complications in these patients, and can assist and inform clinicians throughout the rehabilitation process.

The objective of this review is to describe some of the ways in which EEGs can contribute to clinical practice in pediatric acquired brain injury, in the hope that this will encourage more extensive use of this tool. We present the key literature demonstrating the potential clinical utility of different electrographic biomarkers in clinical populations (adults and children), as well as their use as markers for cognitive and behavioral dysfunction. We note that most of the literature has been gathered from patients with TBI, presumably because this is the most frequent ABI etiology. Moreover, there is considerably more literature on adult and adolescent patients than children.

In Section 2, we present ways in which EEGs are currently used in the critical care setting, including how they are used to monitor and predict epilepsy following TBI, how they can serve as a prognostic marker in assessing the rehabilitative potential in TBI patients, and their use in the evaluation and follow-up of cognitive dysfunction and psychiatric disorders after TBI. In Section 3, we describe the use of EEGs during the rehabilitation of motor disability with a focus on EEG-based brain–computer interfaces as a substitute for impaired motor control. In Section 4, we conclude with a consideration of the factors that will promote the use of EEGs in clinical pediatric settings. Finally, in Section 5, we conclude with a brief consideration of the significant potential of EEG monitoring as an objective physiological biomarker.

## 2. Utility of EEG Monitoring During Rehabilitation

### 2.1. Using EEG to Monitor and Predict Epilepsy After TBI

A common negative outcome of brain injury is the development of epileptic seizures. Post-traumatic seizures are typically categorized based on their timing [19,20]: (1) immediate seizures, occurring less than 24 h after injury; (2) early seizures, occurring between 24 h and one week after injury; and (3) late seizures, occurring more than a week after injury. Post-traumatic epilepsy (PTE) is defined as the occurrence of one or more unprovoked late seizures following TBI [20].

The published incidence of early seizures after TBI in children reaches up to 42.5% [21], and several risk factors have been identified, including abusive head trauma, younger age (especially under 2 years), intracranial hemorrhage, severe TBI, prolonged loss of consciousness, prolonged post-traumatic amnesia, and depressed or open skull fractures [21]. The prevalence of PTE (late seizures) in children after TBI is estimated to range between 10 and 20% [22]; its prevalence is associated with the severity of the injury (e.g., based on the Glasgow Coma Scale (GCS) [19] or neuroimaging findings [23]). However, not much is known regarding the development of PTE, and its relationship to the presence of early seizures, with studies finding mixed results [24]. Some of this uncertainty may stem from the fact that PTE is typically characterized solely based on the occurrence of clinical seizures, without taking into account sub-clinical seizures and other epileptic features (e.g., paroxysmal events) that are clearly visible in patient EEGs, even if they do not manifest as full-blown seizures. It is highly likely that more prolonged EEG monitoring in these patients would reveal a higher prevalence of PTE and provide crucial insight into the epileptogenesis progression [19].

**Figure 2:**
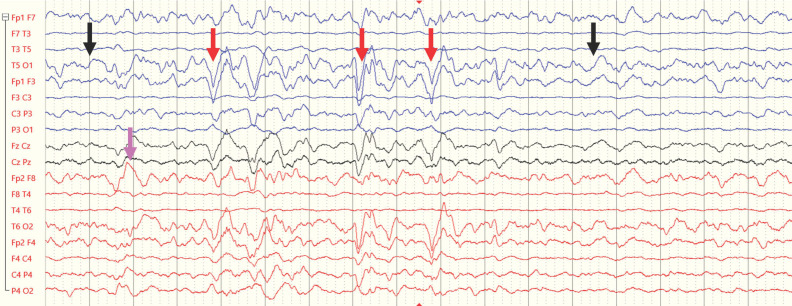
Example EEG recordings from a 4-year-old child during the subacute period following ABI (traces from electrodes over the right and left hemispheres are presented in red and blue, respectively). This example illustrates several abnormal EEG features that are typical in epilepsy but also observed in ABI patients, including increases in the delta/theta power ratio (indicated by black arrows), sporadic epileptiform discharges (indicated by red arrows), and focal polymorphic slowing (indicated by purple arrows).

Studies that have incorporated EEG-based measures as a means of identifying PTE, though initially unsuccessful [25], were able to identify several electrographical markers that are reliable risk factors for PTE among adult patients after TBI. These include increases in the delta/theta power ratio and overall power in this range [25], sporadic epileptiform discharges, and focal polymorphic slowing during the acute period following TBI [26] (Figure 2). Another multi-center study [27] using qEEG measures showed that greater epileptiform activity, delta asymmetry, and lateralized rhythmic delta activity were associated with an increased risk of unprovoked seizures in adults. Recently, Guerriero et al. [28] identified a novel pattern termed macro-periodic oscillations (MOs), a distinct slow periodic pattern of oscillations every 2 to 5 min, which is significantly associated with an increased risk of electrographic seizures in children admitted to the ICU.

These findings clearly demonstrate the potential of EEGs to uncover neural abnormalities and epileptic-like activity that may not be visible to clinicians based solely on behavioral data, and may lead to the under-detection and inadequate treatment of PTE. There is a critical need to characterize these electrographic biomarkers in TBI patients and monitor their development from the acute to the chronic stages of TBI and throughout rehabilitation. This will contribute to developing clinical guidelines, for example, regarding the administration of anti-epileptic medication, which can substantially facilitate patient rehabilitation and reduce the likelihood of clinical seizures [29].

### 2.2. Using EEGs as Prognostic Markers in Assessing Rehabilitative Potential in TBI Patients

Another potential clinical use of EEGs in TBI patients which has gained increased interest in recent years is its use for identifying rehabilitation needs, designing customized interventions, and predicting long-term clinical outcomes in patients with disorders of consciousness following TBI [15]. This is motivated by the fact that current clinical assessment scales, administered upon admission, may aid in assessing the current status of patients, but have limited predictive power regarding more long-term clinical outcomes [15]. Based on studies demonstrating a relationship between specific EEG features and clinical outcomes and prognosis [15,30,31,32,33,34,35,36,37], substantial efforts have been invested in developing an integrative approach that combines clinical assessments with features of patient qEEGs, which can offer greater prognostic value and complement traditional clinical assessments [38].

In reviewing the literature, we can distinguish between EEG-based features identified as potential markers for predicting both positive and negative outcomes in unconscious TBI patients. Several markers proposed as carrying positive prognostic value can be observed in continuous EEGs during sleep. These include increased Slow Wave Activity (SWA) [30] as well as the presence of sleep features such as vertex waves, sleep spindles, and K complexes [32], which have been reported in TBI patients prior to their functional recovery. Others have pointed to global features, such as the degree of EEG reactivity, variability, spectral makeup, and functional connectivity, as predicting positive long-term outcomes in patients with both traumatic and non-traumatic ABI [31,33,39].

Other EEG markers have been associated with negative outcomes. In particular, the dominance of low-frequency activity in the delta and theta range, relative to the higher alpha/beta frequency, has been associated in several studies with poor recovery outcomes [31,33,34]. Similarly, reduced variability in global EEG metrics, such as complexity and entropy, have been associated with poor autonomic physiological function and outcomes [35,36].

However, despite ample work in this field, it is still difficult to pinpoint which specific EEG-based markers carry the most reliable and prognostic value. This is partially because most studies are retrospective and rely on single-time EEG records rather than ongoing, long-term monitoring, limiting our understanding of their adaptability and change throughout recovery. Some have also suggested that considering multiple EEG measures within an individual can substantially improve their collective prognostic power, paving a way forward for their incorporation into clinical care protocols [37,40].

### 2.3. Using EEG to Evaluate Cognitive Dysfunction

Although many children with moderate to severe TBI ultimately regain consciousness, have adequate motor control, and can become independent in basic Activities of Daily Living (ADL, e.g., self-care), they often have persistent difficulties in cognitive (e.g., attentional) and emotional/behavioral functioning [41]. These difficulties severely limit the child’s ability to re-integrate into ‘normative’ life activities, such as going to school and participating in social interactions.

Evaluating cognitive function following TBI is complex due to significant variability in patient ages and developmental background, severity of injury, anatomical location, and underlying mechanisms. Moreover, the currently available metrics used to evaluate and monitor patients’ cognitive/behavioral abilities rely on standardized neuropsychological evaluations, which lack sufficient sensitivity and accuracy, are subjective, and provide insufficient insight into the underlying neuropathology that requires treatment [3,4,5,6]. There is a dire need for objective, sensitive, and quantifiable metrics that capture the severity of the neural pathology and draw a reliable link between specific patterns of pediatric TBI pathologies and their behavioral/cognitive consequences [42,43].

Some attempts have been made to use EEG-based measures as additional metrics of cognitive abilities, focusing both on qEEG resting-state metrics [44] as well as event-related responses (ERPs) to specific stimuli (both with and without a task) [45,46,47,48]. Building on the vast existing knowledge regarding metrics of cognition in the ‘healthy’ brain, many of these studies use well-established paradigms assessing specific cognitive faculties—such as memory, lexical retrieval, inhibitory control, and selective attention—to evaluate the degree to which neural responses in TBI patients resemble those observed in healthy controls. A few studies have even pointed to specific EEG-based metrics that predict recovery of cognitive abilities several years post-injury [49,50].

However, although this line of research may be promising, it suffers from crucial limitations that need to be addressed before we can identify and adopt valid tools that can be used as reliable assessments of an individual’s cognitive abilities or potential cognitive recovery.

One significant limitation, noted above for other measures as well, is the reliance on single EEG measurements rather than repeated evaluations over time, which hinders the ability to assess an individual’s progress during rehabilitation. Additional complicating factors, particularly when attempting to measure ERPs using computerized tasks, include the dependency on an individual’s state of alertness and level of cooperation, and the fact that many ERP metrics do not currently have sufficiently detailed norms at the level of individual participants, a limitation that is particularly challenging in pediatric populations where responses may be age-dependent. Moreover, many studies do not adequately account for factors affecting EEG recordings, such as motor impairments, skull deformities, intraventricular shunts, or epileptic activity. Moreover, there is the fact that EEGs recorded in clinical settings (i.e., in a patient’s hospital room) may suffer from access artifacts, relative to EEGs recorded from healthy controls in highly controlled lab environments [10].

These challenges should not necessarily discourage researchers and clinicians from attempting to use EEGs to identify metrics that reflect cognitive functioning. In fact, we believe that EEGs can be particularly useful for cognitive assessments in individuals who have limited behavioral/motor capacity but may retain cognitive faculties. Indeed, we highlight the factors that need to be considered and dealt with when using EEGs in clinical settings, and encourage collaborations between clinicians and cognitive neuroscientists, whose joint efforts can lead to carefully designed studies and metrics that can be used to assess the cognitive abilities of individual patients and monitor their progress throughout post-TBI rehabilitation.

### 2.4. Using EEGs to Monitor Psychiatric Disorders After TBI

TBI is often accompanied by the development of psychiatric conditions, which severely impacts the patient’s quality of life, and remains an almost constant burden prohibiting full rehabilitation and integration into ‘normative’ social and educational activities. Several studies [51,52,53,54] provided compelling evidence that children who sustain a TBI, including mild traumatic brain injuries (mTBIs), are at a substantially increased risk of developing affective disorders such as depression, anxiety, and adjustment disorders later on in their development. For example, Delmonico et al. [51] found that children with mTBIs were 25% more likely to be diagnosed with an affective disorder compared to those without mTBIs.

The specific nature of the psychiatric complications following pediatric TBI can depend greatly on the patient’s age at the time of injury. For example, a recent study has explored the potential links between an early history of acquired brain injury and the development of secondary autistic spectrum disorder (ASD) in children, showing that children who experience ABI are more likely to be diagnosed with autism later in life [55]. Furthermore, Singh et al. [56] demonstrated that not only do TBI and ASD present similar symptoms, but they also share common underlying biological pathways, suggesting a potential common ground in their etiology. These findings align with the broader consensus that severe early TBI can have profound long-term neurodevelopmental consequences and underscore the vulnerability of the developing brain and the long-term psychological impact that even mild brain injuries can have on children [57].

While numerous studies address the prevalence of mood disorders among patients, they often lack detailed data on unique clinical characteristics and responses to medical treatment. Specifically, from our clinical experience, it is often very difficult to diagnose the specific psychiatric condition or needs of a particular patient, since symptoms often diverge from those described in clinical guidelines [58]. In many cases, it is clinically difficult to determine whether specific symptoms are related to the underlying neurological damage or to psychiatric conditions. Recently, some attempts have been made to use EEGs in order to identify features related to specific neuropsychiatric or neurocognitive conditions and differentiate between them [59,60]. Although still in their infancy, these types of efforts may prove to be extremely useful for clinical purposes, improving early diagnosis of mood and psychiatric disorders, dissociating them from non-psychiatric cognitive deficits, and ultimately designing personalized treatment interventions [61,62].

## 3. Using EEGs During the Rehabilitation of Motor Disability Due to ABI

Besides the cognitive, behavioral, and psychiatric difficulties resulting from ABI, perhaps one of the most debilitating consequences of ABI is motor disability. Motor disabilities can manifest in various forms, including weakness (hemiparesis), paralysis (hemiplegia), spasticity, and coordination problems. These impairments are often the result of damage to motor pathways, including the corticospinal tract, basal ganglia, and cerebellum, which are critical for voluntary motor control. The extent and nature of motor deficits depend on the severity and location of the brain injury [2,61,63]; however, in most cases, they significantly impact patients’ ability to perform everyday activities and consequently their quality of life, as well as that of their caregivers.

### Brain–Computer Interface (BCI)

Besides its use for measuring brain activity, technological advances in recent years have shown that the EEG signal can be effectively used to control assistive devices, such as robotic limbs or computer cursors, constituting a brain–computer interface (BCI). This innovative approach opens up new ways for patients with severe motor impairments to interact with their environment and regain some degree of independence, despite their disability. BCI-based assistive technology was conceived to either augment or replace existing neural rehabilitation methods or to control assistive devices directly through brain activity [64,65,66,67], and its benefits for the rehabilitation of individuals with motor impairments has been demonstrated for enhancing both physical and cognitive performance [68,69]. As BCI applications continue to improve and expand, the EEG signal can now be used to control increasingly complex devices, including virtual reality environments [70], video games [71], and driving humanoid robots [72,73], thus expanding the repertoire of activities that individuals with motor impairment can participate in, despite their disability.

A key element for incorporating EEG-based BCI in the rehabilitative process of children with motor impairments is whether they can successfully learn to operate them. However, research has shown that school-age children are quite proficient in operating simple EEG-based BCI systems and can learn to drive a remote-control car or move a computer cursor using their brainwaves [74,75]. Notably, even children with significant motor and other impairments can use these BCI systems with comparable skill to their typically developing peers [76]. Specific elements that have been shown to improve successful BCI use, particularly in children, are the use of engaging age-appropriate tasks, gamification techniques, and offering suggestions for specific mental-imagery techniques [77,78]; see Saha et al. [79] for a recent review of the challenges and opportunities related to implementing BCI.

Although considerable progress in BCI technology has been made over the past 14 years, BCI for wheelchair-powered mobility is currently still limited for use in highly controlled laboratory environments and is not yet a viable technology for use in clinical settings (see the recent review by Wang et al. [80]). This is partially due to equipment costs, but also to the need for improving algorithms for real-life EEG analysis. Nonetheless, given BCI’s tremendous potential to become a vital access technology for people with severe motor impairments, all research efforts to increase its clinical applicability are encouraged.

## 4. Applicability of EEG Monitoring for Pediatric Rehabilitation of TBI

Previous studies on EEG monitoring in children with acute brain injuries indicate that prolonged or repeated EEG monitoring during the acute phase of the injury reveals electrographic seizure activity in up to 42% of cases, including subclinical seizures [81]. Furthermore, the identification of electrographic seizures improves the ability to predict the clinical course of the patient and enhances understanding of the severity of the brain injury [81,82]. However, there is a significant lack of studies on EEG monitoring during the subacute and chronic phases of brain injury, as well as a notable scarcity of therapeutic protocols for these stages. In order to implement EEG monitoring during the rehabilitation period, the rehabilitation hospital must be equipped with dedicated monitors, a skilled technical team proficient in performing EEG tests, and a pediatric neurologist trained in interpreting the results.

In the past five years at the ALYN rehabilitation center, we have been conducting periodic repeated EEG tests on patients following acquired brain injuries, and we are currently summarizing the results. It is already apparent that the incidence of epileptic disorders in the first years following the injury is very high, as well as the frequency of other abnormal phenomena such as paroxysmal slowing. This finding has dramatically changed our clinical approach to these patients, including early, preventive anti-epileptic medication and avoidance of medications which could exacerbate seizure activity (e.g., stimulants or anti-psychotic drugs). In some cases, we have seen improvement in behavioral symptoms as a result of anti-epileptic treatment. The neurological team has formulated protocols implementing periodic EEG follow-up with periodic psychological assessment.

## 5. Conclusions

The primary objective in rehabilitating children following acquired brain injury is to support reintegration into educational and social environments by restoring the highest level of motor mobility that will lead to their greater independence in basic and instrumental activities of daily living functions. Achieving these goals necessitates stable health, including the management of epileptic complications and improvement in cognitive and meta-cognitive abilities such as attention, judgment, and overall mental and emotional stability.

This review underscored the role and utility of EEG monitoring in diagnosing and tracking the various complications observed in TBI patients. Our findings suggest that EEG monitoring holds significant potential as an objective physiological biomarker that can provide valuable insights into both cognitive and motor functioning for physicians and therapists throughout the rehabilitation process.

Our goal in this review was to raise awareness of the need for such monitoring and the necessity for further research in this area. Most importantly, we aimed to emphasize the critical need for developing therapeutic protocols based on the findings. However, there remains a critical need for further research, particularly longitudinal studies and comprehensive data analytics, to fully understand the associations between the key factors that link ABI severity and recovery potential and to leverage the benefits of EEG monitoring throughout rehabilitation.

## Data Availability

There is no data to be shared in this review article.

## References

[1] Coulter I.C., Forsyth R.J. (2019). Paediatric traumatic brain injury. Curr. Opin. Pediatr..

[2] Popernack M.L., Gray N., Reuter-Rice K. (2015). Moderate-to-Severe Traumatic Brain Injury in Children: Complications and Rehabilitation Strategies. J. Pediatr. Health Care.

[3] Weil Z.M., Karelina K. (2019). Lifelong consequences of brain injuries during development: From risk to resilience. Front. Neuroendocrinol..

[4] Königs M., Heij H.A., van der Sluijs J.A., Vermeulen R.J., Goslings J.C., Luitse J.S., Poll-Thé B.T., Beelen A., van der Wees M., Kemps R.J. (2015). Pediatric Traumatic Brain Injury and Attention Deficit. Pediatrics.

[5] Max J.E., Schachar R.J., Levin H.S., Ewing-Cobbs L., Chapman S.B., Dennis M., Saunders A., Landis J. (2005). Predictors of secondary attention-deficit/hyperactivity disorder in children and adolescents 6 to 24 months after traumatic brain injury. J. Am. Acad. Child Adolesc. Psychiatry.

[6] Le Fur C., Câmara-Costa H., Francillette L., Opatowski M., Toure H., Brugel D., Laurent-Vannier A., Meyer P., Watier L., Dellatolas G. (2020). Executive functions and attention 7 years after severe childhood traumatic brain injury: Results of the Traumatisme Grave de l’Enfant (TGE) cohort. Ann. Phys. Rehabil. Med..

[7] Maloney K.A., Schmidt A.T., Hanten G.R., Levin H.S. (2020). Executive dysfunction in children and adolescents with behavior disorders and traumatic brain injury. Child Neuropsychol..

[8] Narad M.E., Kennelly M., Zhang N., Wade S.L., Yeates K.O., Taylor H.G., Epstein J.N., Kurowski B.G. (2018). Secondary Attention-Deficit/Hyperactivity Disorder in Children and Adolescents 5 to 10 Years After Traumatic Brain Injury. JAMA Pediatr..

[9] Schachar R.J., Park L.S., Dennis M. (2015). Mental Health Implications of Traumatic Brain Injury (TBI) in Children and Youth. J. Can. Acad. Child Adolesc. Psychiatry.

[10] Benedetti G.M., Guerriero R.M., Press C.A. (2023). Review of Noninvasive Neuromonitoring Modalities in Children II: EEG, qEEG. Neurocrit. Care.

[11] Bagnato S., Boccagni C., Prestandrea C., Sant’Angelo A., Castiglione A., Galardi G. (2010). Prognostic value of standard EEG in traumatic and non-traumatic disorders of consciousness following coma. Clin. Neurophysiol..

[12] Nuwer M. (1997). Assessment of digital EEG, quantitative EEG, and EEG brain mapping: Report of the American Academy of Neurology and the American Clinical Neurophysiology Society. Neurology.

[13] Lew H.L., Poole J.H., Guillory S.B., Salerno R.M., Leskin G., Sigford B. (2006). Persistent problems after traumatic brain injury: The need for long-term follow-up and coordinated care. J. Rehabil. Res. Dev..

[14] Amico F., Koberda J.L. (2023). Quantitative Electroencephalography Objectivity and Reliability in the Diagnosis and Management of Traumatic Brain Injury: A Systematic Review. Clin. EEG Neurosci..

[15] Di Gregorio F., La Porta F., Petrone V., Battaglia S., Orlandi S., Ippolito G., Romei V., Piperno R., Lullini G. (2022). Accuracy of EEG Biomarkers in the Detection of Clinical Outcome in Disorders of Consciousness after Severe Acquired Brain Injury: Preliminary Results of a Pilot Study Using a Machine Learning Approach. Biomedicines.

[16] O’Donnell A., Pauli R., Banellis L., Sokoliuk R., Hayton T., Sturman S., Veenith T., Yakoub K.M., Belli A., Chennu S. (2021). The prognostic value of resting-state EEG in acute post-traumatic unresponsive states. Brain Commun..

[17] Engemann D.A., Raimondo F., King J.R., Rohaut B., Louppe G., Faugeras F., Annen J., Cassol H., Gosseries O., Fernandez-Slezak D. (2018). Robust EEG-based cross-site and cross-protocol classification of states of consciousness. Brain.

[18] Chennu S., Annen J., Wannez S., Thibaut A., Chatelle C., Cassol H., Martens G., Schnakers C., Gosseries O., Menon D. (2017). Brain networks predict metabolism, diagnosis and prognosis at the bedside in disorders of consciousness. Brain.

[19] Pease M., Gupta K., Moshé S.L., Correa D.J., Galanopoulou A.S., Okonkwo D.O., Gonzalez-Martinez J., Shutter L., Diaz-Arrastia R., Castellano J.F. (2024). Insights into epileptogenesis from post-traumatic epilepsy. Nat. Rev. Neurol..

[20] Lowenstein D.H. (2009). Epilepsy after head injury: An overview. Epilepsia.

[21] Arndt D.H., Goodkin H.P., Giza C.C. (2016). Early Posttraumatic Seizures in the Pediatric Population. J. Child Neurol..

[22] Strazzer S., Pozzi M., Avantaggiato P., Zanotta N., Epifanio R., Beretta E., Formica F., Locatelli F., Galbiati S., Clementi E. (2016). Late Post-traumatic Epilepsy in Children and Young Adults: Impropriety of Long-Term Antiepileptic Prophylaxis and Risks in Tapering. Paediatr. Drugs.

[23] Xu T., Yu X., Ou S., Liu X., Yuan J., Huang H., Yang J., He L., Chen Y. (2017). Risk factors for posttraumatic epilepsy: A systematic review and meta-analysis. Epilepsy Behav..

[24] Rao V.R., Parko K.L. (2015). Clinical approach to posttraumatic epilepsy. Semin. Neurol..

[25] Pease M., Elmer J., Shahabadi A.Z., Mallela A.N., Ruiz-Rodriguez J.F., Sexton D., Barot N., Gonzalez-Martinez J.A., Shutter L., Okonkwo D.O. (2023). Predicting posttraumatic epilepsy using admission electroencephalography after severe traumatic brain injury. Epilepsia.

[26] Kim J.A., Boyle E.J., Wu A.C., Cole A.J., Staley K.J., Zafar S., Cash S.S., Westover M.B. (2018). Epileptiform activity in traumatic brain injury predicts post-traumatic epilepsy. Ann. Neurol..

[27] Chen Y., Li S., Ge W., Jing J., Chen H.Y., Doherty D., Herman A., Kaleem S., Ding K., Osman G. (2023). Quantitative epileptiform burden and electroencephalography background features predict post-traumatic epilepsy. J. Neurol. Neurosurg. Psychiatry.

[28] Guerriero R.M., Morrissey M.J., Loe M., Reznikov J., Binkley M.M., Ganniger A., Griffith J.L., Khanmohammadi S., Rudock R., Guilliams K.P. (2022). Macroperiodic Oscillations Are Associated With Seizures Following Acquired Brain Injury in Young Children. J. Clin. Neurophysiol.

[29] Maas A.I.R., Menon D.K., Manley G.T., Abrams M., Åkerlund C., Andelic N., Aries M., Bashford T., Bell M.J., Bodien Y.G. (2022). Traumatic brain injury: Progress and challenges in prevention, clinical care, and research. Lancet Neurol..

[30] Mouthon A.L., Meyer-Heim A., Kurth S., Ringli M., Pugin F., van Hedel H.J.A., Huber R. (2017). High-Density Electroencephalographic Recordings During Sleep in Children and Adolescents With Acquired Brain Injury. Neurorehabil. Neural Repair.

[31] Wang J., Huang L., Ma X., Zhao C., Liu J., Xu D. (2022). Role of Quantitative EEG and EEG Reactivity in Traumatic Brain Injury. Clin. EEG Neurosci..

[32] Sandsmark D.K., Kumar M.A., Woodward C.S., Schmitt S.E., Park S., Lim M.M. (2016). Sleep Features on Continuous Electroencephalography Predict Rehabilitation Outcomes After Severe Traumatic Brain Injury. J. Head Trauma Rehabil..

[33] Liuzzi P., Grippo A., Campagnini S., Scarpino M., Draghi F., Romoli A., Bahia H., Sterpu R., Maiorelli A., Macchi C. (2022). Merging Clinical and EEG Biomarkers in an Elastic-Net Regression for Disorder of Consciousness Prognosis Prediction. IEEE Trans. Neural Syst. Rehabil. Eng..

[34] Leon-Carrion J., Martin-Rodriguez J.F., Damas-Lopez J., Barroso y Martin J.M., Dominguez-Morales M.R. (2009). Delta-alpha ratio correlates with level of recovery after neurorehabilitation in patients with acquired brain injury. Clin. Neurophysiol..

[35] Sarà M., Pistoia F. (2010). Complexity loss in physiological time series of patients in a vegetative state. Nonlinear Dyn. Psychol. Life Sci..

[36] Fingelkurts A.A., Fingelkurts A.A., Bagnato S., Boccagni C., Galardi G. (2011). Life or death: Prognostic value of a resting EEG with regards to survival in patients in vegetative and minimally conscious states. PLoS ONE.

[37] Tolonen A., Särkelä M.O.K., Takala R.S.K., Katila A., Frantzén J., Posti J.P., Müller M., van Gils M., Tenovuo O. (2018). Quantitative EEG Parameters for Prediction of Outcome in Severe Traumatic Brain Injury: Development Study. Clin. EEG Neurosci..

[38] Fingelkurts A.A., Fingelkurts A.A., Bagnato S., Boccagni C., Galardi G. (2016). Long-term (six years) clinical outcome discrimination of patients in the vegetative state could be achieved based on the operational architectonics EEG analysis: A pilot feasibility study. Open Neuroimaging J..

[39] Frohlich J., Crone J.S., Johnson M.A., Lutkenhoff E.S., Spivak N.M., Dell’Italia J., Hipp J.F., Shrestha V., Ruiz Tejeda J.E., Real C. (2022). Neural oscillations track recovery of consciousness in acute traumatic brain injury patients. Hum. Brain Mapp..

[40] Ballanti S., Campagnini S., Liuzzi P., Hakiki B., Scarpino M., Macchi C., Oddo C.M., Carrozza M.C., Grippo A., Mannini A. (2022). EEG-based methods for recovery prognosis of patients with disorders of consciousness: A systematic review. Clin. Neurophysiol..

[41] Kohler M.J., Hendrickx M.D., Powell-Jones A., Bryan-Hancock C. (2020). A Systematic Review of Cognitive Functioning After Traumatic Brain Injury in Individuals Aged 10–30 Years. Cogn. Behav. Neurol..

[42] Kim N., Watson W., Caliendo E., Nowak S., Schiff N.D., Shah S.A., Hill N.J. (2022). Objective neurophysiologic markers of cognition after pediatric brain injury. Neurol. Clin. Pract..

[43] Shah S.A., Lowder R.J., Kuceyeski A. (2020). Quantitative multimodal imaging in traumatic brain injuries producing impaired cognition. Curr. Opin. Neurol..

[44] Stein A., Thorstensen J.R., Ho J.M., Ashley D.P., Iyer K.K., Barlow K.M. (2024). Attention Please! Unravelling the Link Between Brain Network Connectivity and Cognitive Attention Following Acquired Brain Injury: A Systematic Review of Structural and Functional Measures. Brain Connect..

[45] Chiang H.S., Motes M., Afkhami-Rohani B., Adhikari A., LoBue C., Kraut M., Cullum C.M., Hart J. (2024). Verbal retrieval deficits due to traumatic brain injury are associated with changes in event related potentials during a Go-NoGo task. Clin. Neurophysiol..

[46] Vanbilsen N., Kotz S.A., Rosso M., Leman M., Triccas L.T., Feys P., Moumdjian L. (2023). Auditory attention measured by EEG in neurological populations: Systematic review of literature and meta-analysis. Sci. Rep..

[47] Campbell A.M., Elbogen E.B., Johnson J.L., Hamer R.M., Belger A. (2021). Event related potentials indexing the influence of emotion on cognitive processing in veterans with comorbid post-traumatic stress disorder and traumatic brain injury. Clin. Neurophysiol..

[48] Larson M.J., Farrer T.J., Clayson P.E. (2011). Cognitive control in mild traumatic brain injury: Conflict monitoring and conflict adaptation. Int. J. Psychophysiol..

[49] Thatcher R.W., Walker R.A., Gerson I., Geisler F.H. (1989). EEG discriminant analyses of mild head trauma. Electroencephalogr. Clin. Neurophysiol..

[50] Thornton K.E. (2003). The electrophysiological effects of a brain injury on auditory memory functioning: The QEEG correlates of impaired memory. Arch. Clin. Neuropsychol..

[51] Delmonico R.L., Tucker L.Y., Theodore B.R., Camicia M., Filanosky C., Haarbauer-Krupa J. (2024). Mild Traumatic Brain Injuries and Risk for Affective and Behavioral Disorders. Pediatrics.

[52] Esterov D., Witkowski J., McCall D.M., Weaver A.L., Brown A.W. (2023). Long-Term Risk for Mood and Anxiety Disorders After Pediatric Traumatic Brain Injury: A Population-Based, Birth Cohort Analysis. J. Head Trauma Rehabil..

[53] Roberts H., Ford T.J., Karl A., Reynolds S., Limond J., Adlam A.R. (2022). Mood Disorders in Young People With Acquired Brain Injury: An Integrated Model. Front. Hum. Neurosci..

[54] Laliberté Durish C., Pereverseff R.S., Yeates K.O. (2018). Depression and Depressive Symptoms in Pediatric Traumatic Brain Injury: A Scoping Review. J. Head Trauma Rehabil..

[55] Porter M., Sugden-Lingard S., Brunsdon R., Benson S. (2023). Autism Spectrum Disorder in Children with an Early History of Paediatric Acquired Brain Injury. J. Clin. Med..

[56] Singh R., Turner R.C., Nguyen L., Motwani K., Swatek M., Lucke-Wold B.P. (2016). Pediatric Traumatic Brain Injury and Autism: Elucidating Shared Mechanisms. Behav. Neurol..

[57] Keenan H.T., Clark A., Holubkov R., Ewing-Cobbs L. (2023). Longitudinal Developmental Outcomes of Infants and Toddlers With Traumatic Brain Injury. JAMA Netw. Open.

[58] Nuckols C.C. (2013). The Diagnostic and Statistical Manual of Mental Disorders, (DSM–5).

[59] Font-Clos F., Spelta B., D’Agostino A., Donati F., Sarasso S., Canevini M.P., Zapperi S., La Porta C.A.M. (2021). Information Optimized Multilayer Network Representation of High Density Electroencephalogram Recordings. Front. Netw. Physiol..

[60] Shor O., Glik A., Yaniv-Rosenfeld A., Valevski A., Weizman A., Khrennikov A., Benninger F. (2021). EEG p-adic quantum potential accurately identifies depression, schizophrenia and cognitive decline. PLoS ONE.

[61] Livint Popa L., Dragos H., Pantelemon C., Verisezan Rosu O., Strilciuc S. (2020). The Role of Quantitative EEG in the Diagnosis of Neuropsychiatric Disorders. J. Med. Life..

[62] Newson J.J., Thiagarajan T.C. (2019). EEG Frequency Bands in Psychiatric Disorders: A Review of Resting State Studies. Front. Hum. Neurosci..

[63] Chevignard M., Câmara-Costa H., Dellatolas G. (2020). Pediatric traumatic brain injury and abusive head trauma. Handb. Clin. Neurol..

[64] Vidal J.J. (1973). Toward direct brain-computer communication. Annu. Rev. Biophys. Bioeng..

[65] Birbaumer N., Ghanayim N., Hinterberger T., Iversen I., Kotchoubey B., Kübler A., Perelmouter J., Taub E., Flor H. (1999). A spelling device for the paralysed. Nature.

[66] Alcaide-Aguirre R., Warschausky S., Brown D., Aref A., Huggins J. (2017). Asynchronous brain-computer interface for cognitive assessment in people with cerebral palsy. J. Neural Eng..

[67] Shahriari Y., Vaughan T.M., McCane L., Allison B.Z., Wolpaw J.R., Krusienski D.J. (2019). An exploration of BCI performance variations in people with amyotrophic lateral sclerosis using longitudinal EEG data. J. Neural Eng..

[68] Lebedev M.A., Nicolelis M.A. (2017). Brain-machine interfaces: From basic science to neuroprostheses and neurorehabilitation. Physiol. Rev..

[69] Saha S., Baumert M. (2020). Intra-and inter-subject variability in EEG-based sensorimotor brain computer interface: A review. Front. Comput. Neurosci..

[70] Vourvopoulos A., Pardo O.M., Lefebvre S., Neureither M., Saldana D., Jahng E., Liew S.L. (2019). Effects of a brain-computer interface with virtual reality (VR) neurofeedback: A pilot study in chronic stroke patients. Front. Hum. Neurosci..

[71] Singh A.K., Wang Y.-K., King J.-T., Lin C.-T. (2020). Extended interaction with a BCI video game changes resting-state brain activity. IEEE Trans. Cogn. Dev. Syst..

[72] Choi B., Jo S. (2013). A low-cost EEG system-based hybrid brain-computer interface for humanoid robot navigation and recognition. PLoS ONE.

[73] Spataro R., Chella A., Allison B., Giardina M., Sorbello R., Tramonte S., Guger C., La Bella V. (2017). Reaching and grasping a glass of water by locked-in ALS patients through a BCI-controlled humanoid robot. Front. Hum. Neurosci..

[74] Zhang J., Jadavji Z., Zewdie E., Kirton A. (2019). Evaluating if children can use simple brain computer interfaces. Front. Hum. Neurosci..

[75] Jadavji Z., Zewdie E., Kelly D., Kinney-Lang E., Robu I., Kirton A. (2022). Establishing a clinical brain-computer interface program for children with severe neurological disabilities. Cureus.

[76] Myrden A., Chau T. (2015). Effects of user mental state on EEG-BCI performance. Front. Hum. Neurosci..

[77] Skola F., Tinkov’a S., Liarokapis F. (2019). Progressive Training for motor imagery brain-computer interfaces using gamification and virtual reality embodiment. Front. Hum. Neurosci..

[78] Buccilli B. (2024). Exploring new horizons: Emerging therapeutic strategies for pediatric stroke. Exp. Neurol..

[79] Saha S., Mamun K.A., Ahmed K., Mostafa R., Naik G.R., Darvishi S., Khandoker A.H., Baumert M. (2021). Progress in Brain Computer Interface: Challenges and Opportunities. Front. Syst. Neurosci..

[80] Wang H., Yan F., Xu T., Yin H., Chen P., Yue H., Chen C., Zhang H., Xu L., He Y. (2021). Brain-Controlled Wheelchair Review: From Wet Electrode to Dry Electrode, from Single Modal to Hybrid Modal, from Synchronous to Asynchronous. IEEE Access..

[81] Arndt D.H., Lerner J.T., Matsumoto J.H., Madikians A., Yudovin S., Valino H., McArthur D.L., Wu J.Y., Leung M., Buxey F. (2013). Subclinical early posttraumatic seizures detected by continuous EEG monitoring in a consecutive pediatric cohort. Epilepsia.

[82] Tewarie P.K., Beernink T.M., Eertman-Meyer C.J., Cornet A.D., Beishuizen A., van Putten M.J., Tjepkema-Cloostermans M.C. (2023). Early EEG monitoring predicts clinical outcome in patients with moderate to severe traumatic brain injury. NeuroImage Clin..

